# *Ampelopsis japonica* Extract Exhibited Significant Uric Acid-Lowering Effect by Downregulating URAT1/GLUT9 and Alleviates Inflammation Through TLR4/NF-*κ*B Pathway

**DOI:** 10.3390/ijms26188999

**Published:** 2025-09-16

**Authors:** Fen Liu, Bai-Lin Li, Meilan Liu, Shaohua Chen, Yaodan Wu, Aikebaier Jumai, Liyun Zhao, Sheng-Xiang Qiu

**Affiliations:** 1 Program for Natural Product Chemical Biology, Key Laboratory of National Forestry and Grassland Administration on Plant Conservation and Utilization in Southern China, South China Botanical Garden, Chinese Academy of Sciences, Guangzhou 510650, China; sixpoints0518@163.com (F.L.); libailin@scbg.ac.cn (B.-L.L.); l15660855546@163.com (M.L.); chensh@scbg.ac.cn (S.C.); wuyaodan@scbg.ac.cn (Y.W.); aikebaier@scbg.ac.cn (A.J.); 2 University of Chinese Academy of Sciences, Beijing 100049, China

**Keywords:** hyperuricemia, metabolomics, network pharmacology, UPLC-QTOF/MS, *Ampelopsis japonica* extract

## Abstract

Hyperuricemia (HUA) is a metabolic disorder characterized by abnormal purine metabolism within the body. *Ampelopsis japonica* (Thunb.) Makino has traditionally been utilized in the treatment of various kidney diseases; however, its specific anti-hyperuricemic effects and the underlying mechanisms warrant further investigation. This study investigates the mechanism of action by which *A. japonica* extract (AJE) addresses HUA using a combination of pharmacology techniques, including network pharmacology and metabolomics. A HUA mouse model was established using potassium oxonate and hypoxanthine. AJE intervention significantly reduced serum uric acid and creatinine levels in HUA mice and markedly decreased glomerular atrophy and renal tubular degeneration. Metabolic profiling revealed distinct metabolic profiles between AJE-intervention and control groups, further demonstrating that AJE corrected disruptions in arginine biosynthesis, purine metabolism, pyrimidine metabolism, and arachidonic acid metabolism. The results of the network pharmacology-based study indicate that AJE can alleviate HUA by modulating the TNF pathway and the Toll-like receptor pathway. The mechanisms of action of AJE in HUA involve the inhibition of xanthine oxidase (XOD) to reduce uric acid synthesis, downregulation of URAT1 and GLUT9 to decrease uric acid reabsorption, and suppression of the TLR4/NF-*κ*B pathway to mitigate inflammation in the HUA mouse model. Therefore, AJE demonstrates significant potential as a therapeutic intervention for HUA and its associated renal complications.

## 1. Introduction

Hyperuricemia is a metabolic disorder characterized by elevated uric acid levels resulting from impaired purine metabolism and abnormal uric acid metabolism [1], making it a key factor in the development of gout. Epidemiological studies conducted in China indicate that the overall prevalence of hyperuricemia is 15%, positioning it as a rapidly growing metabolic disease alongside diabetes, hypertension, and hyperlipidemia [2,3]. Uric acid is a product of purine metabolism, and its dynamic balance in the body is maintained through both production and excretion [4]. An excessive synthesis of uric acid and/or abnormal excretion leads to elevated uric acid levels, accompanied by various pains and inflammations [5]. The production of uric acid primarily occurs in the liver, where xanthine oxidase (XOD) serves as the key enzyme [6] that oxidizes hypoxanthine and xanthine to produce uric acid. With the assistance of urate transport-related proteins [7], both the kidneys and intestines are responsible for the clearance of urate. The kidneys excrete over 70% of uric acid through two types of transporters [8], which are categorized into two major cooperative functional units [9]. One type consists of uric acid reabsorption transporters, including URAT1 [10] and GLUT9, while the other type comprises uric acid secretion transporters, such as ABCG2 [11].

Thus, targeting the synthesis or transport processes of uric acid represents a viable strategy for developing treatments for hyperuricemia [6]. Currently, the management of HUA primarily relies on pharmacological interventions, which involve administering medications [12] to reduce uric acid levels and restore them to the normal range. The three commonly used classes of uric acid-lowering drugs [7] in contemporary treatment include (1) those that reduce uric acid synthesis (e.g., Febuxostat, Allopurinol); (2) those that enhance uric acid excretion (e.g., Benzbromarone, Probenecid); and (3) those that regulate uric acid metabolic hydrolysis (e.g., recombinant Aspergillus flavus urate oxidase and pegylated recombinant urate oxidase). Although these medications effectively lower serum uric acid levels [13], their side effects may limit their clinical application [6]. Traditional Chinese medicine and natural products [14] possess distinct advantages in treating hyperuricemia, as they can modulate human metabolism through multiple pathways and targets, effectively lowering uric acid levels with minimal side effects. Therefore, to develop more effective drugs for anti-hyperuricemia, it is essential to explore the target mechanisms of active components in natural products and verify the various mechanisms exhibited by these components.

The Chinese herbal medicine ‘Bai Lian’ was first documented in the ‘Shennong Ben Cao Jing’ as the tuberous root of *Ampelopsis japonica* (Thunb.) Makino. It is included in the medicinal materials and decoction pieces of the 2025 edition of the ‘Chinese Pharmacopoeia’ Part One. The ‘Shennong Ben Cao Jing’ describes ‘Bai Lian’ as primarily treating abscesses, sores and carbuncles, dispersing stagnant qi, relieving pain [15], and possessing effects such as clearing heat and detoxifying, reducing abscesses and dispersing nodules, and alleviating pain [16]. Notably, the clinical symptoms of damp-heat impediment in traditional medicine closely correspond to those of gouty arthritis induced by modern hyperuricemia [17]. Modern pharmacological research indicates that components such as physcion, chrysophanol, fumaric acid, and gallic acid found in Ampelopsis japonica exhibit antibacterial and anti-inflammatory effects. Additionally, the polysaccharide components demonstrate significant immune-enhancing effects, while the polyphenolic components show notable antioxidant properties. Nutmakul [18] used quercetin as a dietary supplement for hyperuricemia and gouty arthritis. Spinasterol [19] alleviates hyperuricemia-induced renal tubular inflammatory injury and improves renal excretory function by inhibiting NLRP3 inflammasome activation. In this study, we established the uric acid-lowering effects of AJE in a hyperuricemic animal model and unraveled the underlying mechanisms by integrating metabolomic analysis with network pharmacology.

## 2. Results

### 2.1. Main Active Ingredient Analysis of AJE

**Figure 1:**
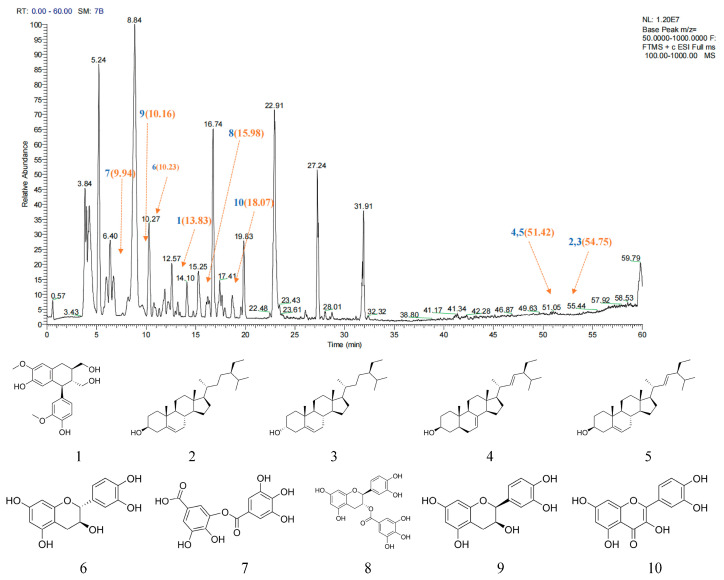
Chemical fingerprint of EtOH crude extract. Blue numbers represent identified compounds. The position of peaks corresponding to compounds was indicated. By orange arrows and number (retention time).

In the present study, HPLC/Q-TOF-MS was used to quantify the components of AJE in both positive-ion and negative-ion modes throughout a retention period of 0–60 min (Figure 1). In the present study ten main active constituents were identified (Table 1) by reference to the ingredients of AJE summarized in the TCMSP (Traditional Chinese Medicine Systems Pharmacology Database and Analysis Platform), including four terpenoids [beta-sitosterol (**2**), sitosterol (**3**), Spinasterol (**4**) and Stigmasterol (**5**)], four flavonoids [(+)-catechin (**6**), (−)-Catechin gallate (**8**), ent-Epicatechin (**9**) and quercetin (**10**)], a phenolic acid [digallate (**7**)] and (2R,3R,4S)-4-(4-hydroxy-3-methoxy-phenyl)-7-methoxy-2,3-dimethylol-tetralin-6-ol (**1**).

### 2.2. Intersection of the Targets Between AJE and Hyperuricemia

**Figure 2:**
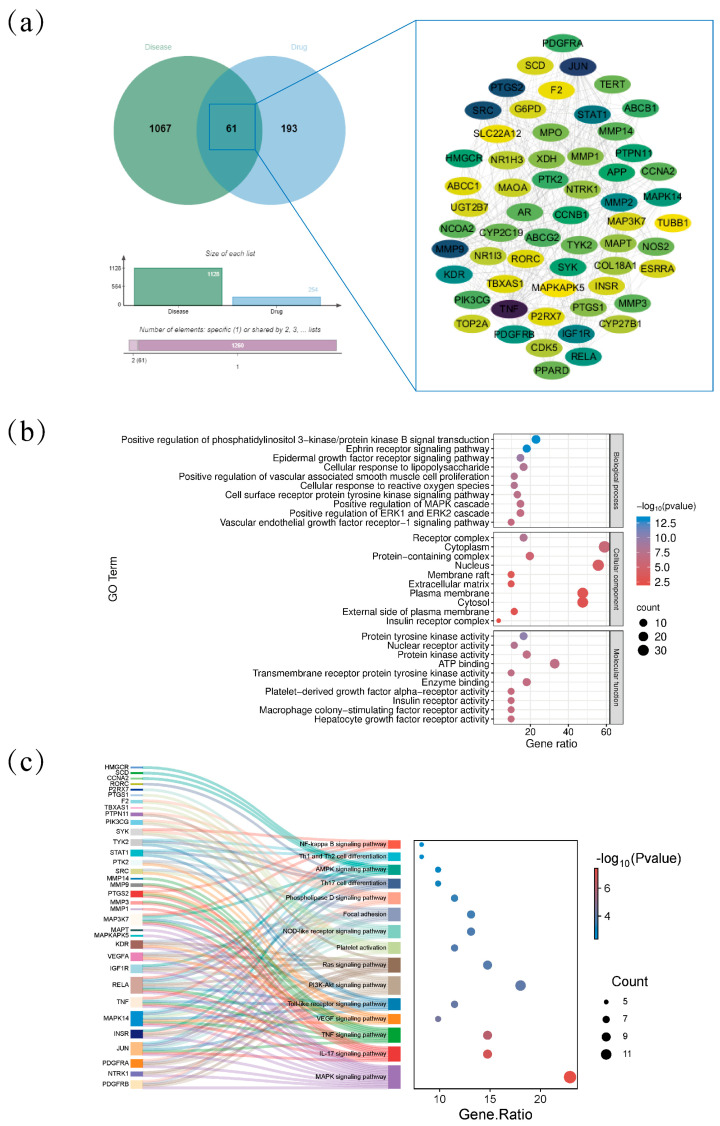
Network pharmacology analysis on the association between *Ampelopsis japonica* on HUA. (**a**) Protein–protein interaction (PPI) network of HUA targets by AJE. Enrichment analysis results of AJE against HUA according to adjusted *p*-value and counts screening, from GO Ontology enrichment analysis (**b**) and the top 15 pathways from KEGG enrichment analysis (**c**).

Network pharmacology was used to examine the responsible active ingredients and mechanism of action of AJE in the treatment of hyperuricemia. Based on the screening parameters, ten active components were identified. The TCMSP database evaluated 166 component targets. After searching the Gene Cards and OMIM databases, removing duplicate entries, and taking the union of the results, a total of 1128 protein targets related to HUA were identified. Potential targets for the treatment of hyperuricemia were identified by intersecting 166 component targets with 1128 hyperuricemia-related targets to provide 61 pharmacological targets (Figure 2a). The intersection targets were imported into the STRING database to construct a protein–protein interaction (PPI) network, using node degree as the evaluation parameter. A higher node degree indicates greater importance of the node within the network. The size of the node and the darkness of its color correspond to the number of targets connected to it. In the protein–protein interaction (PPI) network, target proteins with higher degrees include TNF, JUN, PTGS2, MMP9, SRC, STAT1, IGF1R, MMP2, KDR, PDGFRB, and RELA.

BP, CC, and MF were among the 61 important nodes that were imported into the Metascape platform for GO enrichment analysis (Figure 2b). Of these, the BP was primarily implicated in the cytokine-mediated signal pathway, aging, and positive control of the apoptotic process. The macromolecular complex and nucleoplasm contained the majority of the CC. The identical protein binding and enzyme binding were the primary foci of the MF. In the meantime, the Metascape platform was used to import the 61 critical nodes mentioned above for KEGG pathway analysis (Figure 2c). The findings showed that it was primarily involved in MAPK signaling pathway, IL-17 signaling pathway, TNF signaling pathway, VEGF signaling pathway, Toll-like receptor signaling pathway and PI3K-Akt signaling pathway, which may be the main targets for hyperuricemia treatment.

### 2.3. Effects of AJE on Serum UA, CRE, BUN, and Body Weight in Hyperuricemia Mice

**Figure 3:**
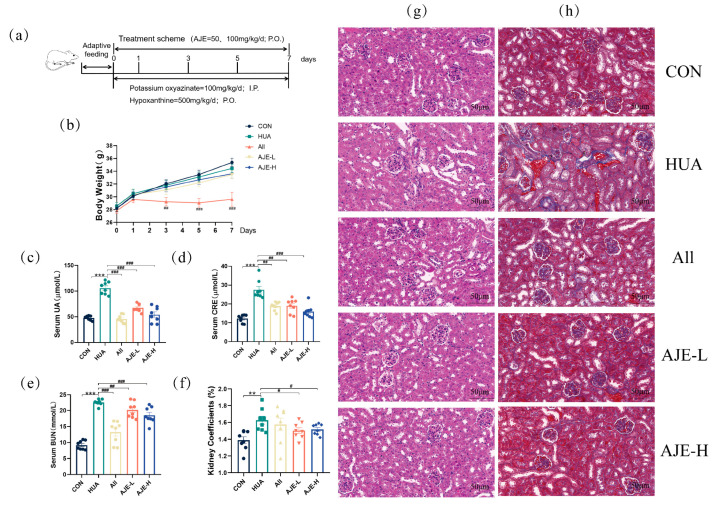
Effects of AJE in HUA-affected mice on important physiological markers. CON stood for the group of healthy mice. A mouse group dosed with 500 mg/kg/d of hypoxanthine and 100 mg/kg/d of potassium oxyzinate to produce HUA was referred to as HUA. Group for Allopurinol (All) was used as a positive treatment. After the model was established, the All, AJE-L, and AJE-H were administered one hour later (*n* = 8). (**a**) The dose regimen. (**b**) The weight fluctuation throughout a seven-day period. (**c**) The serum uric acid levels in the indicated groups. (**d**) The serum creatinine levels in the indicated groups. (**e**) The serum blood urea nitrogen levels in the indicated groups. (**f**) Kidney index for the groups listed. (**g**,**h**) Sample slices stained with H&E (**g**) and Masson (**h**) from the kidneys of the respective groups. Scale bar = 50 μm, original magnification 400×. Values represent mean ± standard error of the mean. * *p* < 0.05, ** *p* < 0.01 and *** *p* < 0.001 versus the CON group. ^#^ *p* < 0.05, ^##^ *p* < 0.01 and ^###^ *p* < 0.001 versus the HUA group.

The experimental protocol (Figure 3a) which conformed to established standards, involved the oral administration of AJE to HUA mice at daily doses of 50 and 100 mg/kg. The animal dosages were adjusted to human equivalents using body surface area calculations. Although the HUA-induced group had marginally lower body weights compared to the CON group (Figure 3b), this difference was not statistically significant. However, a notable reduction in body weight was observed in the HUA group following AJE prevention. Compared to the control group, the body weight of mice significantly decreased following allopurinol administration. This reduction may be attributed to common side effects such as gastrointestinal discomfort, as well as the stress and metabolic burden induced by the modeling process itself. The interplay of these factors likely exacerbates the observed effects. Serum levels of UA, CRE and BUN are pivotal for evaluating HUA and renal function, respectively. The HUA model group showed significantly higher levels of UA (105.9 ± 11.95 μmol/L), Cre (22.57 ± 4.95 μmol/L) and BUN (22.58 ± 0.96 mmol/L) compared to the CON group (Figure 3c–e), confirming the validity of the model. Crucially, AJE-L group and AJE-H group preventions led to significant decreases in UA levels (67.44 ± 7.58; 54.37 ± 14.94 μmol/L) in the improved mice compared to the hyperuricemia group. Meanwhile, AJE-L group and AJE-H group preventions led to significant decreases in Cre levels (19.08 ± 3.65; 15.98 ± 3.31 μmol/L) and BUN levels (20.21 ± 2.49; 18.52 ± 2.51 mmol/L) in the improved mice compared to the hyperuricemia group.

### 2.4. Effects of AJE on Kidney Injury in Hyperuricemia Mice

The kidney size in the HUA group was significantly larger than that in the CON group (*p* < 0.01), indicating the presence of inflammation and kidney damage in the HUA mice (Figure 3f and Figure S2). After prevention with AJE, however, kidney size became closer to that of the CON group, suggesting a repair in kidney damage. Kidneys from the HUA group displayed characteristic pathological alterations, including glomerular atrophy and degeneration, vacuolar degeneration of renal tubular epithelium cellular swelling, and dilated lumens (Figure 3g). Additionally, the HUA group showed bluer Masson staining in the kidney compared to the CON group, indicating greater collagen buildup, which is a marker of tissue scarring (Figure 3h). Preventions with AJE and a known urate-lowing medication allopurinol (All) led to significantly reduced collagen accumulation in the kidneys of the HUA mice. These findings suggested that AJE may ameliorate kidney fibrosis and inflammation in hyperuricemia mice.

### 2.5. AJE Ameliorates HUA-Induced Renal Inflammation

**Figure 4:**
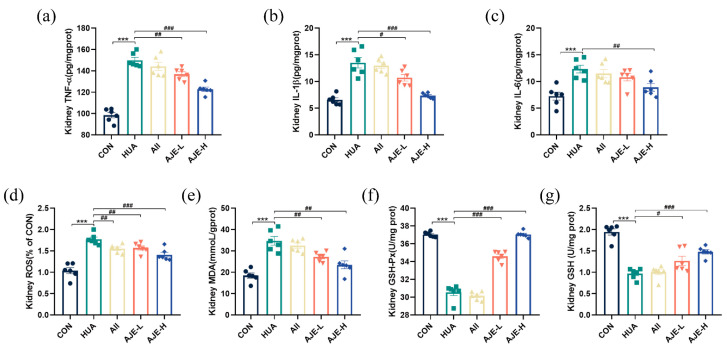
Effect of AJE on HUA induced kidney oxidative stress and inflammation. (**a**) The kidney TNF-α levels in the experimental groups (*n* = 6). (**b**) The kidney IL-1β levels in the experimental groups (*n* = 6). (**c**) The kidney IL-6 levels in experimental groups (*n* = 6). (**d**) The kidney ROS levels in the experimental groups (*n* = 6). (**e**) The kidney MDA levels in the experimental groups (*n* = 6). (**f**) The kidney GSH-Px levels in the experimental mice groups (*n* = 6). (**g**) The kidney GSH levels in the experimental mice groups (*n* = 6). Mean ± standard error of the mean is shown by values. * *p* < 0.05, ** *p* < 0.01 and *** *p* < 0.001 versus the CON group. ^#^ *p* < 0.05, ^##^ *p* < 0.01 and ^###^ *p* < 0.001 versus the HUA group.

Excess uric acid can induce oxidative stress and inflammation in the kidney. As expected, the concentrations of multiple inflammatory cytokines including tumor necrosis factor-alpha (TNF-α), interleukin-1 beta (IL-1β) and interleukin-6 (IL-6) were higher in the kidney of the hyperuricemia group than those in the CON group, indicating the presence of inflammation (Figure 4a–c). However, prevention with both doses of AJE (AJE-L and AJE-H) significantly lowered the levels of TNF-α, IL-1β and IL-6 in the kidney of the hyperuricemic mice (*p* < 0.01). Furthermore, the content of ROS and malondialdehyde (MDA) was significantly increased in the kidneys of the hyperuricemic mice, while the activities of the glutathione peroxidase (GSH-Px) and glutathione (GSH) were significantly decreased in the kidneys of the hyperuricemic mice (Figure 4d–g). Compared to the HUA group, however, AJE prevention resulted in a decrease in MDA (*p* < 0.01) content and a significant increase in the activities of GSH-Px (*p* < 0.01) and GSH (*p* < 0.05). This evidence supported the hypothesis that AJE can mitigate oxidative stress and protect the kidneys in hyperuricemic mice.

### 2.6. XOD Inhibitory Activity of AJE

**Figure 5:**
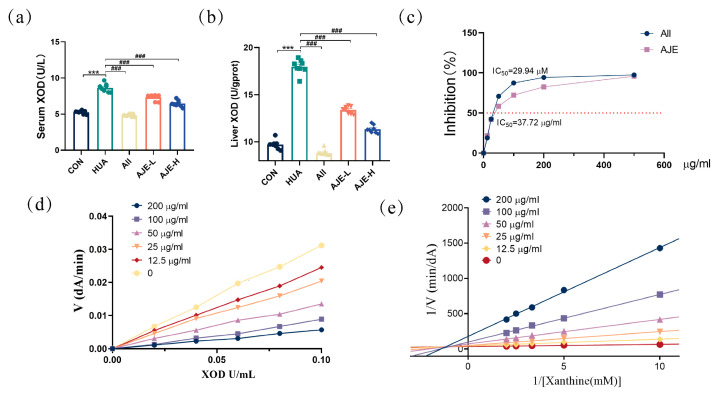
The effect of AJE on liver injury and xanthine oxidase. (**a**) Serum XOD levels in the designated groups (*n* = 8). (**b**) Liver XOD levels in the designated groups (*n* = 8). (**c**) Inhibitory actions of XOD. (**d**) The AJE’s V-[XO] plots. (**e**) AJE Burk plots using Lineweaver. Values represent mean ± standard error of the mean. * *p* < 0.05, ** *p* < 0.01 and *** *p* < 0.001 versus the CON group. ^#^ *p* < 0.05, ^##^ *p* < 0.01 and ^###^ *p* < 0.001 versus the HUA group.

Elevated serum and liver XOD activity, a sign of metabolic stress from hyperuricemia, was observed in mice. It can also induce hepatocyte damage (Figure S3a–c), leading to the abnormal release of serum transaminases. After AJE treated (Figure 5a,b), a significant reduction in XOD activity was noted, indicating therapeutic alleviation of oxidative stress and enzymatic rebalancing. This decrease likely reflects enhanced purine metabolism and a favorable treatment response in managing hyperuricemia. To explore the potential mechanism by which AJE reduces uric acid level, we assessed its effect on XOD activity in vitro. Allopurinol, a standard inhibitor of XOD, showed the strongest inhibition with an IC_50_ of 29.94 μM. AJE also significantly inhibited XOD activity in a dose-dependent manner, reducing the oxidation of xanthine with an IC_50_ of 37.72 μg/mL (Figure 5c). Experiments confirmed that AJE’s inhibition of XOD was reversible (Figure 5d), as increasing AJE concentrations led to decreased reaction velocities, and each velocity versus [XOD] plot passed through the origin. A double reciprocal analysis indicated that AJE acts as a mixed inhibitor (Figure 5e), mainly showing competitive inhibition. This was evidenced by increased line slopes with rising AJE concentrations and a common intersection in the second quadrant, along with distinct Y-axis intersections for each concentration.

### 2.7. Serum Principal Component Analysis

**Figure 6:**
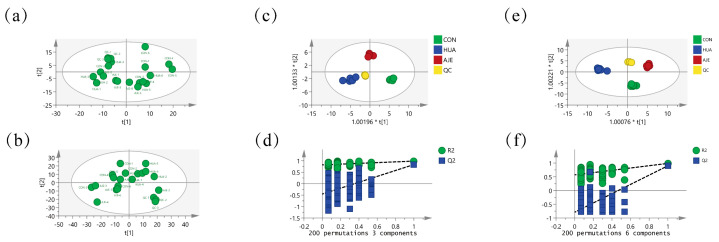
Multivariate statistical analysis. The metabolomics analysis of serum samples is plotted in positive and negative modes by the PCA score (**a**,**b**); in positive and negative modes by the OPLS-DA score (**c**,**e**); in positive and negative modes by the OPLS-DA model validation diagram (**d**,**f**).

To understand how AJE ameliorates HUA in mice, we conducted a metabolomic analysis on blood samples using UPLC-MS/MS. The overall distribution and stability of the analysis process concerning positive and negative ions across the three data groups were evaluated using principal component analysis (PCA). The groups were distinctly separated, while the data within each group exhibited close clustering, with minimal overlap and a limited range (Figure 6a,b). The S-plot (Figure S1a,b) indicates that the majority of metabolite ions are concentrated near the origin, while a minority of ions deviate from the origin. All samples fell within the 95% confidence interval, indicating significant differences between the groups. Furthermore, the analytical approach, encompassing both the preprocessing and instrumental analysis methods, demonstrated stability and reliability.

### 2.8. Screening and Identification of Potential Biomarkers

The differences among the three groups of samples were evaluated using the Orthogonal Partial Least Squares Discriminant Analysis (OPLS-DA) model, with results presented in Figure 6c–f. In both positive and negative ion modes, the HUA group and the AJE group were distinctly classified into two categories, indicating significant differences between the two groups. R^2^Y represents the degree of fit of the model to the data, while Q^2^ indicates the model’s predictive ability. The closer R^2^Y and Q^2^ are to 1, the better the model’s performance. A smaller R^2^ and a negative Q^2^, along with an upward trend in the regression line, suggest that the permutation test was successful and that there was no overfitting in the model. The cumulative R^2^Y and Q^2^ of the established positive ion OPLS-DA model were 0.984 and 0.832, respectively, while those of the negative ion model were 0.991 and 0.893, indicating high stability and predictive capability of the model. The results of the 200 permutation tests revealed that the positive ions R^2^ and Q^2^ were 0.82 and −0.452, respectively, while the negative ions R^2^ and Q^2^ were 0.586 and −1.28, respectively, all of which were lower than the actual model values. Therefore, the OPLS-DA model did not exhibit overfitting, demonstrating high reliability.

**Figure 7:**
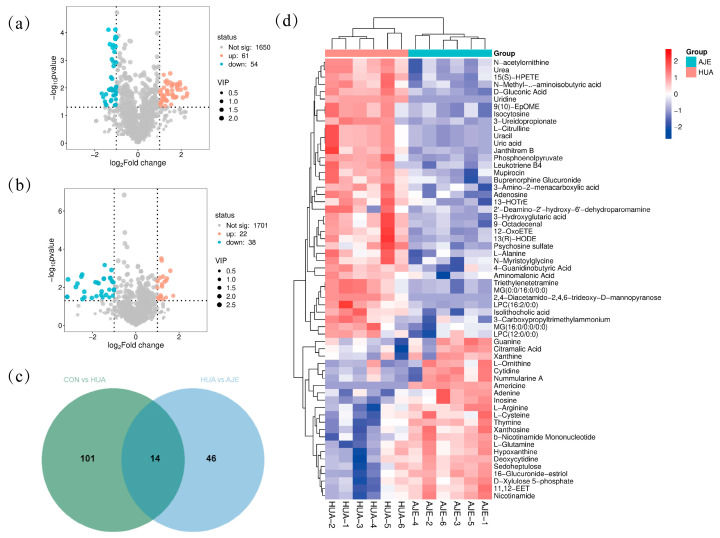
(**a**) Volcanic map of metabolites with significant differences between CON group and HUA group. (**b**) Volcanic map of metabolites with significant differences between HUA group and AJE group. (**c**) Significant differences in metabolites at the intersection between groups. (**d**) Heatmaps of 60 significantly altered metabolites; the darker the color, the greater the abundance value. Compared with the HUA group, blue represents a decrease in differential metabolite content after AJE intervention, while red represents an increase in differential metabolite content after AJE intervention.

The variable importance in projection (VIP) parameters derived from the OPLS-DA analysis were utilized to screen for differential variables between samples, thereby identifying endogenous metabolites with significant contributions. Metabolites exhibiting a VIP > 1, statistical significance (*p* < 0.05), and a fold change > 2 were designated as potential biomarkers. The identification of metabolites was achieved by integrating chromatographic elution information, fragmentation patterns, adduct ionization modes, molecular composition ratios, multi-stage mass spectrometry data, and relevant literature. In comparison to the CON group, the levels of 115 serum metabolites were significantly altered in the HUA group, with 61 metabolites being upregulated and 54 downregulated (Figure 7a). Conversely, when compared to the HUA group, the levels of 60 serum metabolites were significantly altered in the AJE group, with 22 metabolites upregulated and 38 downregulated (Figure 7b). The Venn diagram illustrates the differential metabolites at the intersection between the CON vs. HUA and HUA vs. AJE groups. Notably, 14 metabolites were significantly modulated following AJE intervention (Figure 7c, Table 2). Figure 7d presents a clustering heatmap of 60 differential metabolites. This visualization tool is employed to illustrate the similarities and differences in the expression levels and other characteristics of the metabolites. The differential metabolites were classified into several categories, including amino acids and their derivatives, lipid derivatives, fatty acids and their conjugates, carbohydrates and their conjugates, purines and their derivatives, organic amines, and glycerophospholipids. Among these categories, the first three constituted the majority, likely due to the fact that hyperuricemia can lead to disturbances in amino acid and lipid metabolism.

**Table 2:** Differential metabolites in the callback between CON vs. HUA and HUA vs. AJE groups.

No.	Metabolites	Formula	VIP	Trend	
				CON vs. HUA	HUA vs. AJE
1	Thymine	C_5_H_6_N_2_O_2_	2.11	↓	↑
2	Deoxycytidine	C_9_H_13_N_3_O_4_	1.79	↓	↑
3	Cytidine	C_9_H_13_N_3_O_5_	1.56	↓	↑
4	Xanthosine	C_10_H_12_N_4_O_6_	2.11	↓	↑
5	Adenine	C_5_H_5_N_5_	1.95	↓	↑
6	Xanthine	C_5_H_4_N_4_O_2_	1.38	↓	↑
7	Guanine	C_5_H_5_N_5_O	1.59	↓	↑
8	Hypoxanthine	C_5_H_4_N_4_O	1.71	↓	↑
9	Inosine	C_10_H_12_N_4_O_5_	2.27	↓	↑
10	Adenosine	C_10_H_13_N_5_O_4_	2.22	↑	↓
11	Urea	CH_4_N_2_O	2.37	↑	↓
12	Uracil	C_4_H_4_N_2_O_2_	2.44	↑	↓
13	Uric acid	C_5_H_4_N_4_O_3_	2.44	↑	↓
14	Leukotriene B4	C_20_H_32_O_4_	2.18	↑	↓

“↑” indicates up-regulation, “↓” indicates down-regulation.

### 2.9. Metabolic Pathway Analysis

**Figure 8:**
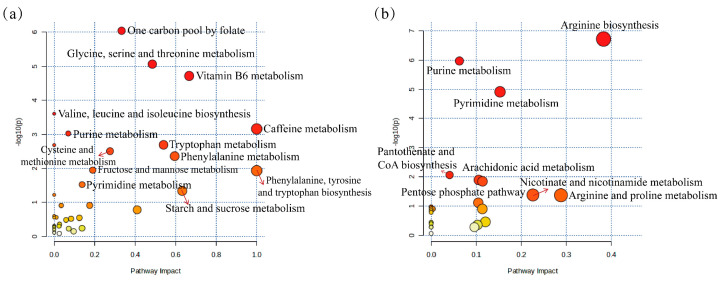
Metabolic pathway analysis. (**a**) Bubble plot analysis of metabolic pathway enrichment between CON group and HUA group. (**b**) Bubble plot analysis of metabolic pathway enrichment between HUA group and AJE group. A metabolic route is represented by each dot. The pathway represented by the circle is described by the text indicated by the red arrow.

Compared to the CON group, the serum levels of 115 differential metabolites in the HUA group were altered. These metabolites were subsequently imported into MetaboAnalyst for further analysis. The onset and progression of hyperuricemia primarily involve metabolic pathways, including the one-carbon pool by folate, glycine, serine, and threonine metabolism, vitamin B6 metabolism, valine, leucine, and isoleucine biosynthesis, purine metabolism, and pyrimidine metabolism (Figure 8a). In comparison to the HUA group, the AJE group effectively intervened in the metabolic disorders of serum metabolites in hyperuricemic mice. The 60 differential metabolites were primarily enriched in pathways associated with arginine biosynthesis, purine metabolism, pyrimidine metabolism, pantothenate and CoA biosynthesis, the pentose phosphate pathway, arachidonic acid metabolism, and nicotinate and nicotinamide metabolism, as well as arginine and proline metabolism (Figure 8b).

### 2.10. The Effect of AJE on the TLR4/NF-κB Pathway in the Renal Tissue of Hyperuricemia Mice

**Figure 9:**
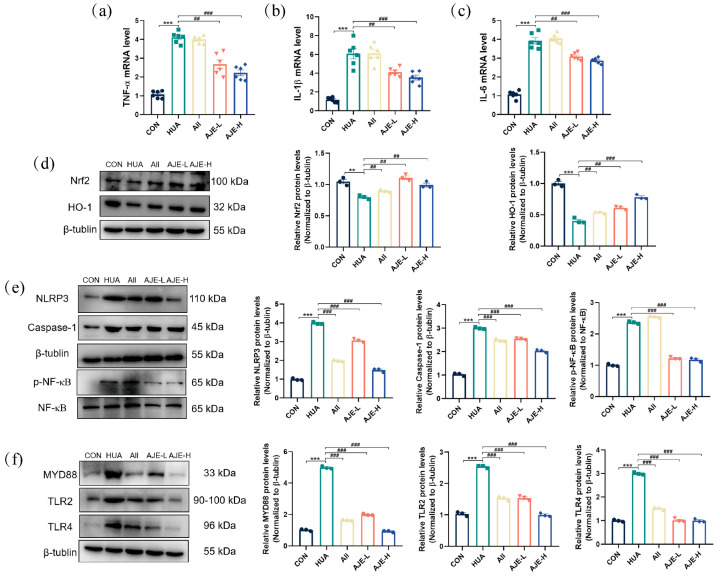
Effects of AJE in hyperuricemic mice on the expression in the TLR4 signaling pathway. (**a**) The kidney expressions of TNF-*α* (*n* = 6) assessed by RT-qPCR. (**b**) The expression of IL-1*β* in the kidney assessed by RT-qPCR (*n* = 6). (**c**) The expression of IL-6 in the kidney assessed by RT-qPCR (*n* = 6). (**d**) The Nrf2 (*n* = 3) and HO-1 (*n* = 3) levels in the kidney assessed by Western blot. (**e**) The levels of NLRP3 (*n* = 3), Caspase-1 (*n* = 3) and p-NF-*κ*B (*n* = 3) proteins in the kidney assessed by Western blot. (**f**) The levels of MyD88 (*n* = 3), TLR2 (*n* = 3) and TLR4 (*n* = 3) proteins in the kidney assessed by Western blot and their analysis. Mean ± standard error of the mean is shown by values. * *p* < 0.05, ** *p* < 0.01 and *** *p* < 0.001 versus the CON group. ^#^ *p* < 0.05, ^##^ *p* < 0.01 and ^###^ *p* < 0.001 versus the HUA group.

In comparison to the CON group, the mRNA levels of inflammatory and stress markers, including IL-1*β*, TNF-*α*, and IL-6 (Figure 9a–c), were approximately twofold higher (*p* < 0.001) in the hyperuricemia group, indicating a significant upregulation of these genes in response to hyperuricemia (HUA). However, prevention with both doses of AJE (AJE-L and AJE-H) significantly reduced the mRNA levels of IL-1*β*, TNF-*α*, and IL-6 (*p* < 0.01).

In hyperuricemic mice, the baseline expression of nuclear factor erythroid 2-related factor 2 (Nrf2) in the nucleus was low due to increased oxidative stress and inflammation associated with elevated uric acid levels. This resulted in reduced activation of downstream antioxidant genes, including HO-1 (*p* < 0.01), indicating a compromised antioxidant defense system. Upon prevention with AJE, Nrf2 expression was upregulated (Figure 9d), along with the expression of antioxidant genes such as HO-1. Furthermore, AJE prevention suppressed the activation of the NLRP3 inflammasome, reducing the assembly of the complex and subsequent activation of Caspase-1 (Figure 9e). AJE also inhibited NF-*κ*B activation by suppressing its phosphorylation (Figure 9e). Compared to the CON group, the HUA group exhibited upregulated protein levels of MyD88, TLR2 and TLR4 (Figure 9f). However, administration of AJE dose-dependently reduced the protein levels of MyD88, TLR2 and TLR4 (*p* < 0.01) (Figure 9f).

### 2.11. AJE Up-Regulated ABCG2 and Down-Regulated GLUT9 and URAT1

**Figure 10:**
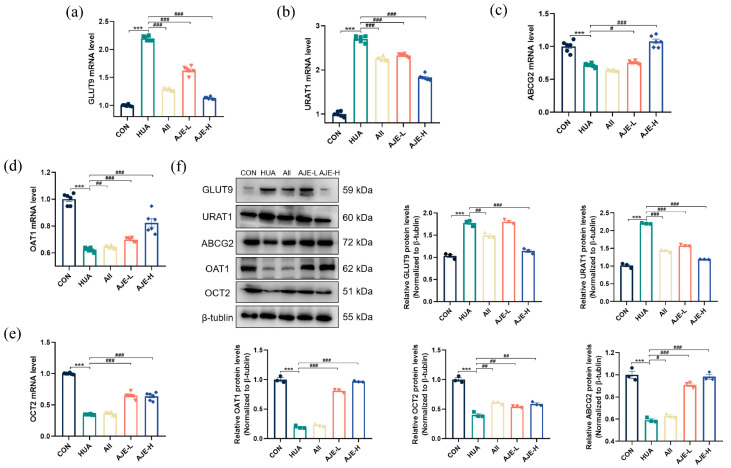
Effects of AJE on the transporters associated with uric acid metabolism in hyperuricemic mice (**a**–**e**). The kidney expression of GLUT9 (**a**), URAT1 (**b**), ABCG2 (**c**), OAT1 (**d**) and OCT2 (**e**) in the indicated mouse groups assessed by RT-qPCR (*n* = 6). (**f**) The kidney levels of GLUT9 (*n* = 3), URAT1 (*n* = 3), ABCG2 (*n* = 3), OAT1 (*n* = 3) and OCT2 (*n* = 3) in the indicated mice groups assessed by Western blot and their analysis. Mean ± standard error of the mean is shown by values. * *p* < 0.05, ** *p* < 0.01 and *** *p* < 0.001 versus the CON group. ^#^ *p* < 0.05, ^##^ *p* < 0.01 and ^###^ *p* < 0.001 versus the HUA group.

In addition to xanthine oxidase, several transporters play crucial roles in the metabolism of uric acid. Two key urate transporters, URAT1 and GLUT9 are primarily involved in the reabsorption of urate in the kidneys, while OAT1, OCT2 and ABCG2 are the three main transporters responsible for the excretion of uric acid. To assess the impact of AJE on these transporters, we utilized RT-qPCR to evaluate the expressions of OAT1, GLUT9, URAT1, OCT2 and ABCG2 (Figure 10a–e) in the experimental mice. The induction of the hyperuricemia model with hypoxanthine and potassium oxonate in mice resulted in a significant decrease in the expression of OAT1, ABCG2 and OCT2 (*p* < 0.001). Prevention with AJE at a dose of 100 mg/kg significantly increased the mRNA levels of OAT1, ABCG2 and OCT2 (*p* < 0.01). Furthermore, while the mRNA levels of GLUT9 and URAT1 were elevated in the model group compared to the normal CON group (*p* < 0.001), both doses of AJE prevention led to a reduction in the expression of GLUT9 and URAT1 (*p* < 0.001). As illustrated in Figure 10f, the HUA group exhibited an upregulation of kidney URAT1 and GLUT9 expression compared to the control group (*p* < 0.001). Conversely, a downregulation of ABCG2, OAT1 and OAT3 was observed in the HUA group relative to the control. These findings suggest that hyperuricemia influences the expression of key kidney transporters involved in uric acid excretion. Notably, AJE was able to reverse these hyperuricemia-induced alterations (*p* < 0.001), indicating that it modulates renal uric acid excretion by affecting both secretion and reabsorption pathways.

## 3. Discussion

Hyperuricemia is not only defined as a disorder of purine metabolism but is also recognized as a pathogenic factor contributing to gout, kidney disease and cardiovascular diseases [5]. This study employed a combination of hypoxanthine and potassium oxonate [20] to induce an acute hyperuricemia model in Kunming male mice: the former serves as a key precursor in purine metabolism, promoting the generation of uric acid, while the latter specifically inhibits uricase activity, thereby blocking the catabolism of uric acid. Certainly, it is important to acknowledge that the research findings may be constrained by the inherent physiological differences between mouse models and human pathophysiology [21]. These differences could, to some extent, impact the efficacy of the drugs. Pharmacodynamic studies have shown that AJE intervention exhibits a dose-dependent regulatory effect on creatinine and blood urea nitrogen levels, as well as on the renal organ coefficient, indicating its distinct renal protective effect. Concurrently, assessments of pathological morphology further confirm that AJE can significantly ameliorate pathological manifestations such as glomerular structural atrophy and inflammatory cell infiltration.

Elevated XOD activity is a key driver of excessive uric acid synthesis [6]. Our study demonstrated that AJE significantly reduced both serum XOD levels in mice with HUA. Therefore, AJE could mitigate hyperuricemia by inhibiting XOD production. In vitro enzyme activity assays, specifically the Lineweaver-Burk plots, revealed that AJE functions as a mixed-type inhibitor of XOD. The intersecting lines on the graph and converging in the second quadrant indicate that AJE preferentially binds to the free enzyme rather than the enzyme-substrate complex, underscoring its potential as an effective regulator of XOD activity.

In our study, we adopted a comprehensive approach, integrating pharmacodynamics, network pharmacology, and metabolomics to investigate the therapeutic effects and mechanisms of AJE in improving hyperuricemia. To uncover the active constituents and pathways through which AJE exerted its effects, we analyzed its chemical composition using chemical fingerprinting and network pharmacology. AJE was found to contain a range of bioactive compounds, including beta-sitosterol, sitosterol, spinasterol, stigmasterol, (+)-catechin gallate, (−)-catechin gallate, and quercetin. These compounds were likely to constitute the active basis through which AJE mitigated hyperuricemia, thereby providing a scientific rationale for its therapeutic potential. The network pharmacology results revealed that among the key proteins targeted by AJE in the prevention of HUA, TNF, JUN, RELA, PTGS2, PTGS1, and NOS2 are closely associated with inflammation [22,23] and oxidative stress. AJE’s therapeutic impact on hyperuricemia might be exerted through the suppression of inflammatory and oxidative stress responses. Network pharmacology-based analyses and predictions rely on existing databases and literature, which may introduce certain biases. Therefore, further in-depth basic experiments are essential for validation. The ELISA test results indicated that AJE concentration-dependently reduced the levels of inflammatory factors TNF-*α*, IL-1*β*, and IL-6 in kidney homogenates, as well as the mRNA expression in kidney tissues. This suggests that AJE can decrease the release of inflammatory factors and possesses anti-inflammatory effects, thereby confirming the predictions made by network pharmacology. Based on these predictions, subsequent experimental validation was conducted on the inflammation-related TLR4/NF-*κ*B pathway to explore the mechanism of AJE in treating HUA. Elevated levels of Nrf2 and HO-1 typically coincide with a reduction in oxidative stress indicators such as MDA and an enhancement in reduced GSH levels [24]. The Nrf2/HO-1 pathway is crucial for the body’s antioxidant defense and significantly mitigates the inflammation and pain associated with hyperuricemia and gout [25]. In the context of hyperuricemia, the NLRP3 inflammasome is often activated in mice due to the presence of uric acid crystals [26], which the immune system recognizes as a danger signal. This activation initiates the assembly of the NLRP3 inflammasome complex, a pivotal step in the inflammatory response. Upon activation, the NLRP3 inflammasome facilitates the cleavage of pro-Caspase-1 into its active form [27], which then leads to the maturation and release of pro-inflammatory cytokines such as IL-1*β* [28]. TLR4, a pattern recognition receptor [29], engages MyD88 to initiate a signaling cascade that results in NF-*κ*B-mediated inflammation when the organism is injured. However, in our study, the expression levels of NLRP3, caspase-1, and TLR4 in renal tissues, were significantly diminished in mice treated with AJE. Western blot tests substantiated the network pharmacology findings, confirming that AJE’s anti-inflammatory and antioxidant effects are indeed closely associated with the Nrf2/HO-1 and TLR4/NF-*κ*B signaling pathways. This validation underscores the potential of AJE as a therapeutic agent in modulating these pathways to alleviate hyperuricemia and its associated inflammation.

Based on the changes in differential metabolites and related metabolic pathways identified through metabolomics, 60 significantly differential metabolites were identified between the AJE high-dose group and the model group. Eight major metabolic pathways were obtained, including those associated with purine metabolism, energy metabolism, oxidative stress, inflammation regulation, and amino acid metabolism [30]. In hyperuricemia, abnormal levels of cytosine and cytidine in pyrimidine metabolism can disrupt the anabolism of phospholipids, leading to an elevated glomerular filtration rate. Additionally, the deamination of cytosine to uracil results in excessively high concentrations of uracil, which can induce abnormal renal hemorheology and further exacerbate kidney damage [31]. Mice with hyperuricemia exhibited elevated levels of 2-deoxycytidine and abnormalities in the pyrimidine metabolic pathway. After prevention with AJE, serum uracil and 2-deoxycytidine levels were normalized, and uric acid levels returned to normal, suggesting that AJE can influence the pyrimidine metabolic pathway by regulating serum uracil levels. Arachidonic acid [32] is an inflammatory fatty acid known to initiate inflammatory responses. Research has shown that abnormal metabolism of arachidonic acid is closely linked to renal inflammation. Furthermore, leukotriene B4, a metabolite of arachidonic acid, is recognized as a significant inflammatory factor [33,34] associated with gout. This study demonstrates that AJE can modulate the arachidonic acid metabolic pathway, reduce the release of the pro-inflammatory factor LTB4, and alleviate inflammation associated with hyperuricemia. These findings confirm that AJE may exert renal protective effects by mitigating inflammatory damage. Xanthine and hypoxanthine regulate XOD activity and decrease purine catabolism [35]. AJE may stabilize purine metabolism indirectly by inhibiting purine and pyrimidine metabolism, balancing the nucleotide pool, and reducing uric acid production.

In the regulatory system of uric acid metabolism, URAT1 and GLUT9 serve as the primary transporters on the renal tubular epithelial cell membrane [36], mediating the active uptake of uric acid from the renal tubular lumen into the cells [37]. Notably, this transport system maintains a dynamic balance with the secretion network composed of ABCG2 [38,39], OAT1 and OCT2 [40]. The former responsible for the reabsorption of uric acid, while the latter promotes the final excretion of uric acid through efflux at the basolateral membrane [26]. Further molecular experimental results indicate that the hyperuricemia mouse model exhibits a bidirectional imbalance in the uric acid transport system: in addition to the overexpression of URAT1 and GLUT9 on the brush border membrane of the proximal tubule, the mRNA and protein levels of basolateral ABCG2, OAT1 and OCT2 are significantly downregulated. AJE not only dose-dependently inhibits the mRNA and protein synthesis of URAT1 and GLUT9 but also restores the functional expression of secretory transporters such as ABCG2. This bidirectional regulatory effect suggests that AJE may achieve its therapeutic effect by reconstructing the dynamic balance of uric acid transport absorption and secretion in the renal tubules.

## 4. Materials and Methods

### 4.1. Reagents and Materials

Hypoxanthine (≥98%, HPLC) and potassium oxonate were purchased from Sigma-Aldrich (St. Louis, MO, USA). Allopurinol (≥99.97%, HPLC) were purchased from MedChemExpress (Shanghai, China). Xanthine oxidase (XOD) kits, creatinine kits and uric acid kits were taken from the Nanjing Jiancheng Bioengineering Institute (Nanjing, China).

The research samples were provided by our own laboratory prepared according to our preliminary research on the phytochenistry of AJE [41], namely, the dried roots of AJ (500 g) were pulverized and refluxed with 4 L of 70% ethanol for 40 min, twice. The pooled filtrates were concentrated under a vacuum to yield a greenish powder, which was dissolved in water and saved for use.

### 4.2. Network Pharmacology Analysis of AJE

The effective chemical components of AJE were gathered using the TCMSP (http://tcmspw.com/tcm-sp.php; accessed on 24 October 2024), a systematic pharmacology database and analysis platform for traditional Chinese medicine, along with the TCMID (http://www.megabionet.org/tcmid/; accessed on 24 October 2024), a comprehensive database for traditional Chinese medicine. A preliminary screening was performed based on the criteria of oral bioavailability ≥ 30% and drug-likeness ≥ 0.18, leading to the identification of potential components of AJE. The targets of AJE were predicted using the TargetNet website (http://targetnet.scbdd.com/; accessed on 24 October 2024). The gene symbol matching the action targets of the AJE component was obtained using the Uniport protein database (https://www.uniprot.org/; accessed on 24 October 2024). To identify disease-related targets of hyperuricemia, search for related human genes in the TTD (https://db.idrblab.org/ttd/; accessed on 24 October 2024), OMIM (http://www.omim.org/; accessed on 24 October 2024), and GeneCards (https://www.genecards.org/; accessed on 24 October 2024) databases. Remove any duplicates and filter the results to exclude targets with a score of 0.5 or higher. Venn diagrams were created to show how AJE action targets and disease targets combine to provide shared potential targets. To assess the protein–protein interaction, common candidate targets were uploaded to the STRING database (https://string-db.org/; accessed on 11 April 2025). The DAVID database (https://david.ncifcrf.gov/; accessed on 11 April 2025) was utilized to conduct enrichment analysis for the KEGG and GO pathways.

### 4.3. Animals and Prevention

Male SPF KM mice (18–24 g) were obtained from the Medical Experimental Animal Center of Guangdong Province (license number: SCXK (YUE) 2018-0002) and kept in a controlled environment with a 12 h light/dark cycle and a room temperature of 21 ± 2 °C, as well as regular access to food and water. All experiments were approved and conducted under the related ethics regulations of the Committee on Use and Care of Animals of the Institute of Biological and Medical Engineering, Guangdong Academy of Sciences (Guangzhou, China) under protocol number and the Guide for the Care and Use of Laboratory Animals (US National Research Council, 1996). The ethics number is K2024-01-122.

Male SPF KM mice were randomly divided into the following five groups (*n* = 8) after one week of adaptive feeding: control (CON), hyperuricemia (HUA), HUA treated with Allopurinol at the dose of 7.6 mg/kg/d, HUA treated with AJE at the dose of 50 mg/kg/d, and HUA treated with AJE at the dose of 100 mg/kg/d. The method reported previously [42] was exploited for establishing the hyperuricemic models by dosing 500 mg/kg/d HX together with 100 mg/kg/d PO for all groups except the CON group. The administration of the drugs was conducted at 1 h after the model establishment [20].

### 4.4. Sample Collection

Before sacrifice, all mice were fasted for 12 h. Mouse serum, liver and kidney were collected following a 7-day experiment. The blood samples were collected from the orbital plexus and centrifuged at 3000 rpm for 15 min at 4 °C to obtain serum for subsequent metabolomics analysis and other biochemical indicators. Simultaneously, the kidney and liver were promptly extracted and cleaned with saline. The partial tissue samples were submerged in a 10% neutral formalin buffer. Samples of liver and kidney were taken from each mouse’s left lateral and instantly snap-frozen in liquid nitrogen.

### 4.5. Histological Examination

After being fixed in 10% formalin solution for 24 h and embedded in paraffin, the kidney samples were sectioned (5 μm) and stained with hematoxylin-eosin (H&E). Transmission electron microscopy was then used to examine the ultrastructure of the kidney tissue for histological evaluation.

### 4.6. Determination of Serum and Organ Tissues Biochemical Indicators

Tissue samples from kidneys were weighed and mixed with precooled 10% saline, then centrifuged at 4 °C, 4000 rpm for 15 min. Serum levels of uric acid (UA), blood urea nitrogen (BUN) and creatinine (Cre) were quantified using commercial kits. The supernatant was analyzed for XOD, reactive oxygen species (ROS) activity, malondialdehyde (MDA), glutathione peroxidase (GSH-Px) activity, and glutathione (GSH) levels, following kit instructions.

### 4.7. Determination of XOD Inhibitory Activity

XOD (0.05 U/mL) and xanthine (0.2 mM) were prepared and stored at 4 °C for later use. AJE was diluted to concentrations of 0, 12.5, 25, 50, 100, and 200 µg/mL with PBS. Blank and positive controls were set up using PBS and allopurinol, respectively. The assay was conducted in 96-well plates, each well containing 100 µL of the reaction mixture. According to the literature methods [43], combined with the exploration of laboratory conditions, AJE (100 µL of varying concentrations) was mixed with 50 µL of XOD and incubated for 10 min at 37 °C. Xanthine (50 µL) was then added to start the reaction, and absorbance at 295 nm was monitored for 10 min to calculate XOD inhibitory activity using the following formula:

$$\mathrm{Inhibition}\;\mathrm{percentage}\;\left(\%\right)=\frac{\left[\left(\mathrm{dA}/\mathrm{dt}\right)\;\mathrm{blank}\;-(\mathrm{dA}/\mathrm{dt})\;\mathrm{sample}\right]}{(\mathrm{dA}/\mathrm{dt})\;\mathrm{blank}},$$

where (dA/dt) blank represents the reaction rate in the absence of an inhibitor, and (dA/dt) sample denotes the rate with various samples, the IC_50_ values were determined from the average data points.

The type of XOD inhibition by AJE was determined using Lineweaver-Burk kinetics, which involved measuring initial reaction rates at varying xanthine concentrations with and without the inhibitor.

### 4.8. Metabolomics Analysis

#### 4.8.1. Serum Sample Preparation

Serum samples were thawed on ice after storage at −80 °C. 50 μL of the sample were combined with 300 μL of extraction solution (acetonitrile:methanol = 1:4) containing internal standards in a 2 mL tube. Following vortexing for 3 min, samples were centrifuged at 300× *g* for 10 min at 4 °C. In total, 200 μL of the supernatant were collected and chilled at −20 °C for 30 min, and were centrifuged again at 300× *g* for 3 min at 4 °C. 180 μL of the supernatant were used for LC-MS analysis.

#### 4.8.2. UPLC-QTOF/MS Analysis

The AJE sample was analyzed using an LC-ESI-MS/MS system (UPLC, ExionLC AD; MS, QTRAP^®^ System; Jasco, Tokyo, Japan) with a 1.8 μm, 2.1 × 100 mm column at 40 °C, a flow rate of 0.4 mL/min, and an injection volume of 2 μL or 5 μL. The solvent system was water (0.1% formic acid) and acetonitrile (0.1% formic acid) with a gradient program. The Triple TOF mass spectrometer acquires MS/MS spectra during LC/MS experiments. Set ESI source parameters: ISVF 5500 V or −4500 V, gas pressures, and source temperature at 500 °C.

#### 4.8.3. Data Processing and Multivariate Statistical Analysis

Metabolite data were analyzed using SIMCA 14.1 version (Umetrics AB, Umea, Sweden) for Principal Component Analysis (PCA) and Orthogonal partial least squares discriminant analysis (OPLS-DA). Differential metabolites were identify with absolute Log2FC (|Log2FC| > 2) and VIP (VIP > 1). MetaboAnalyst R was used for VIP values after mean centering and log transformation. A permutation test was conducted with 200 permutations. Identified metabolites were annotated using the MetaAnalyst database (http://www.metaboanalyst.ca/; accessed on 15 April 2025) and mapped them to the pathway database.

### 4.9. RT-qPCR Analysis

Total RNA from the kidney was extracted using TRIzol reagent (Thermo Fisher Scientific, Waltham, MA, USA) following the manufacturer’s protocol. Using the corresponding kit, 2 μg total RNA was reverse transcribed to cDNA, followed by PCR amplification in the presence of SYBR Green. Primer sequences for the target genes were designed using BLAST (https://blast.ncbi.nlm.nih.gov/Blast.cgi, accessed on 24 October 2024) and synthesized by BGI Gene (Table 3). Gene expression was normalized to *β*-actin, and the relative expression levels were calculated using the 2^−ΔΔCt^ method.

### 4.10. Western Blot Analysis

Kidney tissues were processed for protein extraction using RIPA buffer on ice, with added PMSF, followed by centrifugal separation at 300× *g* for 10 min at 4 °C. The protein concentration was normalized to 5 µg, and equal amounts were loaded onto a 10% SDS-PAGE gel. After electrophoresis, proteins were transferred to a PVDF membrane. Membranes were blocked with 5% skimmed milk in TBST to reduce non-specific binding. They were then incubated with primary antibodies against NLRP3, MyD88, TLR2, TLR4, Caspase-1, ABCG2, URAT1, GLUT9, OAT1, OCT2, Nrf2, HO-1, p-NF-*κ*B, NF-*κ*B and β-tublin overnight at 4 °C, each diluted in TBST. The membranes were subsequently washed with TBST and incubated with HRP-conjugated secondary antibody for 1.5 h at room temperature followed by washing with TBST. Protein bands were detected using an ECL substrate and a Tanon 5200 Multi system (Shanghai, China). Each experiment was repeated three times, and band intensities were measured using Image J software (https://imagej.net/ij/, accessed on 24 October 2024).

### 4.11. Statistical Analysis

The data are presented as means ± standard deviation. The differences among multiple groups were analyzed using one-way ANOVA followed by the LSD test, with *p* < 0.05 considered statistically significant. GraphPad Prism version 8.0 (GraphPad Software Inc., San Diego, CA, USA) was utilized for all statistical analyses. A *p*-value of less than 0.05 was considered statistically significant.

## 5. Conclusions

Administering AJE orally resulted in reduced serum uric acid and creatinine levels in hyperuricemic mice, while also mitigating kidney damage, including glomerular atrophy and tubular cell degeneration. Our study demonstrated that AJE modulates purine metabolism, pyrimidine metabolism, and arachidonic acid metabolism, leading to decreased uric acid levels. Additionally, AJE attenuated kidney inflammation by inhibiting the Nrf2/HO-1 and TLR4/NF-*κ*B signaling pathways. AJE exhibited a significant uric acid-lowering effect through the dual inhibition of uric acid synthesis and absorption by targeting XOD and downregulating URAT1 and GLUT9, respectively. Overall, AJE effectively alleviated the symptoms of hyperuricemia.

## Figures and Tables

**Table 1:** Screening results of active ingredients in AJE.

No.	Molecule	MW	AlogP	Hdon	Hack	OB (%)	DL
AJE-01	(2*R*,3*R*,4*S*)-4-(4-hydroxy-3-methoxy-phenyl)-7-methoxy-2,3-dimethylo	360.44	2.25	4	6	66.51	0.39
AJE-02	Beta-sitosterol	414.79	8.08	1	1	36.91	0.75
AJE-03	Sitosterol	414.79	8.08	1	1	36.91	0.75
AJE-04	Spinasterol	412.77	7.64	1	1	42.98	0.76
AJE-05	Stigmasterol	412.77	7.64	1	1	43.83	0.76
AJE-06	(+)-Catechin	290.29	1.92	5	6	54.83	0.24
AJE-07	Digallate	322.24	1.53	6	9	61.85	0.26
AJE-08	(−)-Catechin gallate	442.40	3.16	7	10	53.57	0.75
AJE-09	ent-Epicatechin	290.29	1.92	5	6	48.96	0.24
AJE-10	Quercetin	302.25	1.5	5	7	46.43	0.28

**Table 3:** PCR primers for the key genes associated with hyperuricaemia.

Description	GenBank	Primer Name	Primer Sequences (5′-3′)	Product Size (bp)	T_m_ (°C)
*Actb*	NM_007393.5	M-*Actb*-F	GATATCGCTGCGCTGGTCG	132	61.37
		M-*Actb*-R	CATTCCCACCATCACACCCT		59.67
*IL-1β*	NM_008361.4	M-*IL-1β*-F	AATGCCACCTTTTGACAGTGA	139	58.33
		M-*IL-1β*-R	GATGTGCTGCTGCGAGATTT		59.27
*TNF-α*	NM_001278601.1	M-*TNF-α*-F	GATCGGTCCCCAAAGGGATG	92	60.18
		M-*TNF-α*-R	CCACTTGGTGGTTTGTGAGTG		59.59
*URAT1*	NM_009203.3	M-*URAT1*-F	TCATCACGGTAGGCAAGCTG	100	60.11
		M-*URAT1*-R	ACCTCCATGGTCAACGTGTC		59.97
*GLUT9*	NM_001012363.2	M-*GLUT9*-F	TGCATTGGCGTGTTTTCTGG	193	59.97
		M-*GLUT9*-R	GGAAGGCTTTCATGGCTCCT		60.03
*ABCG2*	NM_001355477.2	M-*ABCG2*-F	GATGCCGCTGGAATGCAAAA	95	60.11
		M-*ABCG2*-R	ACTACGAACAGCTCCACAGC		60.04
*OAT1*	NM_008766.3	M-*OAT1*-F	CGTCGGAAGGTGCTGATCTT	121	60.11
		M-*OAT1*-R	TAGCCAAAGACATGCCCGAG		60.11
*OAT3*	NM_001164634.2	M-*OAT3*-F	TGGCTACAGTTGTCCGTGTC	133	59.97
		M-*OAT3*-R	TAGCCACACGTTGGAGTGTC		59.97
*IL-6*	NM_001314054.1	M-*IL-6*-F	CACTTCACAAGTCGGAGGCT	355	59.97
		M-*IL-6*-R	GCCACTCCTTCTGTGACTCC		60.04
*OCT2*	NM_001355767.1	M-*OCT2*-F	CAGAATTTGTTGGGCTGGGC	147	60.04
		M-*OCT2*-R	AGAAGTTGGGCAGAGTCACG		59.97

## Data Availability

All data are reported in the published version of this article. Raw data can be made available upon reasonable request to the authors.

## Supplementary Materials

The following supporting information can be downloaded at https://www.mdpi.com/article/10.3390/ijms26188999/s1.

## Abbreviations

The following abbreviations are used in this manuscript:

AJ	*Ampelopsis japonica* (Thunb.) Makino
AJE	*Ampelopsis japonica* extract
ABCG2	ATP-binding cassette subfamily G member 2
BUN	Blood urea nitrogen
Cre	Creatinine
GLUT9	Glucose transporter 9
HUA	Hyperuricemia
HX	Hypoxanthine
NF-*κ*B	Nuclear factor-*κ*B
OAT1	Organic anion transporter protein 1
OPLS-DA	Orthogonal partial least-squares discriminant analysis
PCA	Principal component analysis
PO	Potassium oxonate
TLR4	Toll-like receptor 4
UA	Uric acid
URAT1	Urate transporter 1
UPLC-QTOF/MS	Ultra-performance liquid chromatography-quadruple time-of-flight/mass spectrometry
XOD	Xanthine oxidase
VIP	Variable importance projection
