# Supplementary material for: Ampelopsis japonica Extract Exhibited Significant Uric Acid-Lowering Effect by Downregulating URAT1/GLUT9 and Alleviates Inflammation Through TLR4/NF-κB Pathway
Source: Int J Mol Sci. 2025 Sep 16;26(18):8999. doi: 10.3390/ijms26188999 (PMC12469466; doi:10.3390/ijms26188999)
Supplement: Supplementary file 1 [file ijms-26-08999-s001.zip › ijms-3749815-supplementary.pdf]

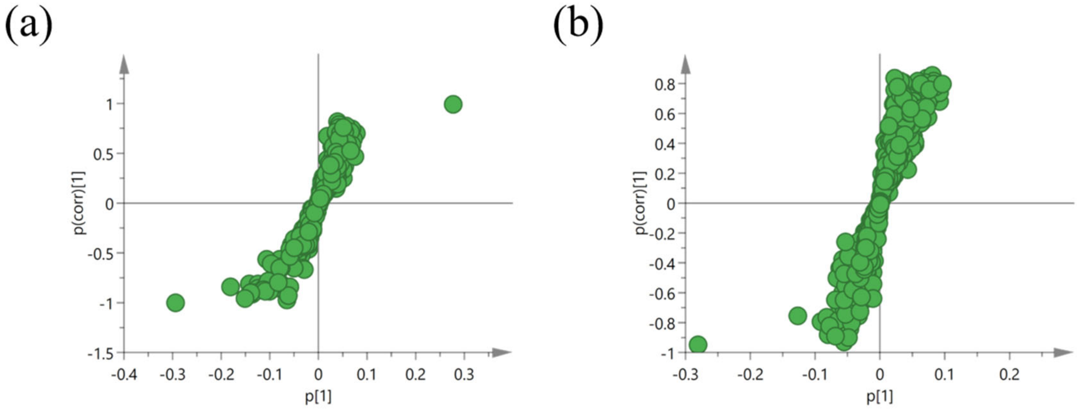

Figure S1 S-plot in metabolomics analysis. (a) S-plot in positive mode metabolomics analysis; (b) S-plot in negative mode metabolomics analysis.

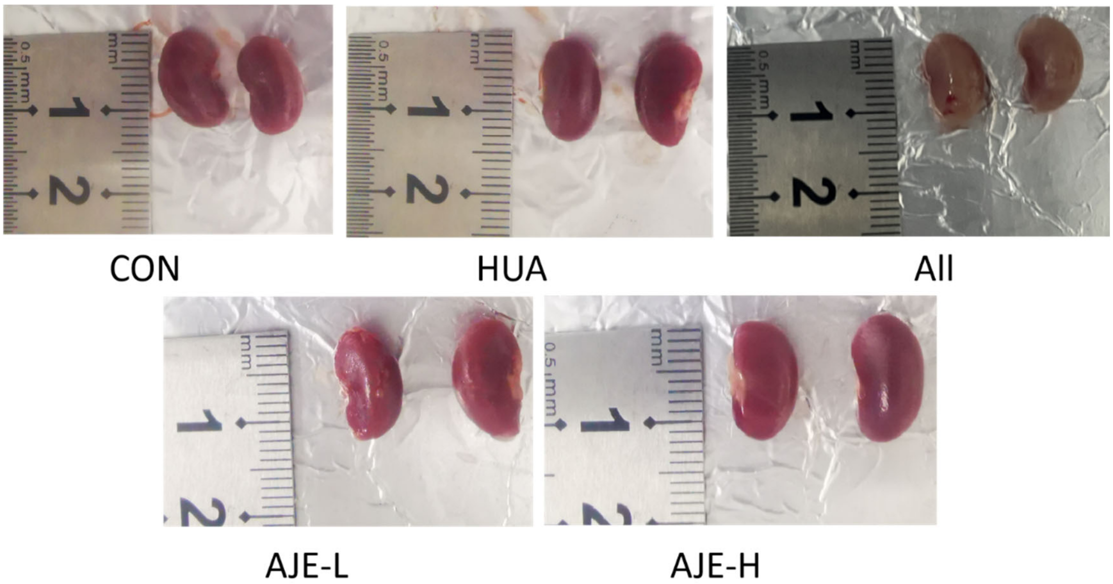

Figure S2 Morphology assessment of the kidney injury in experimental mice. CON mouse group were healthy mice. HUA mouse group were dosing with 100mg/kg/d potassium oxyzinate and 500mg/kg/d hypoxanthine to induce hyperuricemic. All mouse group were allopurinol mice. The administration of the AJE-L and AJE-H was conducted at 1 h after the model establishment (n=8).

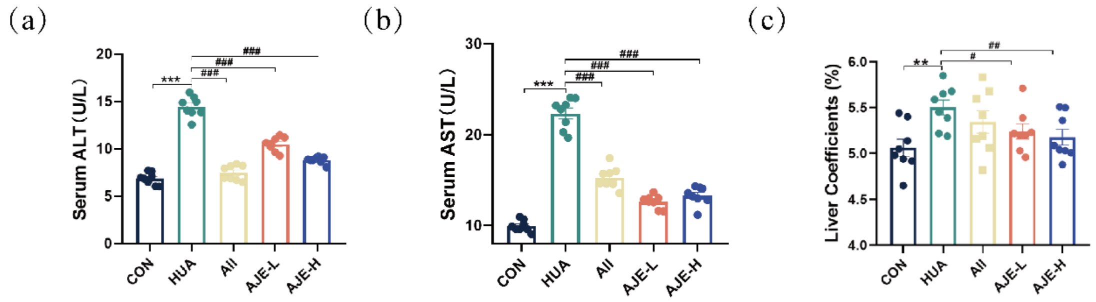

Figure S3 The Effect of AJE on the Liver of Mice with Hyperuricemia. CON mouse group were healthy mice. HUA mouse group were dosing with 100mg/kg/d potassium oxyzinate and 500mg/kg/d hypoxanthine to induce hyperuricemic. All mouse group were allopurinol mice. The administration of the AJE-L and AJE-H was conducted at 1 h after the model establishment (n=8). (a) The serum ALT levels in the indicated groups. (b) The serum AST levels in the indicated groups. (c) Liver index for the groups listed. Values represent mean ± standard error of the mean. $^{*}P<0.05$, $^{**}P<0.01$ and $^{***}P<0.001$ versus the CON group. $^{\#}P<0.05$, $^{\#\#}P<0.01$ and $^{\#\#\#}P<0.001$ versus the HUA group.
